# A step towards 6D WAXD tensor tomography

**DOI:** 10.1107/S2052252524003750

**Published:** 2024-05-09

**Authors:** Xiaoyi Zhao, Zheng Dong, Chenglong Zhang, Himadri Gupta, Zhonghua Wu, Wenqiang Hua, Junrong Zhang, Pengyu Huang, Yuhui Dong, Yi Zhang

**Affiliations:** a Beijing Synchrotron Radiation Facility, Institute of High Energy Physics Chinese Academy of Sciences (CAS) Beijing100049 People’s Republic of China; b Spallation Neutron Source Science Center, Dongguan523803, People’s Republic of China; c School of Engineering and Materials Science Queen Mary University of London LondonE1 4NS United Kingdom; d University of Chinese Academy of Sciences Beijing100049 People’s Republic of China; e Shanghai Synchrotron Radiation Facility, Shanghai Advanced Research Institute Chinese Academy of Sciences Shanghai201204 People’s Republic of China; f Institute of High Energy Physics Chinese Academy of Sciences Beijing100049 People’s Republic of China; g Institute of Biomedical Engineering, Chinese Academy of Medical Sciences and Peking Union Medical College, Tianjin300192, People’s Republic of China; Lund University, Sweden; Keele University, United Kingdom

**Keywords:** computed tomography, wide-angle X-ray diffraction, computational modeling, structure prediction, virtual reciprocal-space scans, 6D tomography, diffraction tensors, voxel reconstruction

## Abstract

X-ray scattering/diffraction tensor tomography techniques are promising methods to acquire the 3D texture information of heterogeneous biological tissues at micrometre resolution. However, the methods suffer from a long overall acquisition time due to multi-dimensional scanning across real and reciprocal space. Here, a new approach is introduced to obtain 3D reciprocal information of each illuminated scanning volume using mathematic modeling, which is equivalent to a physical scanning procedure for collecting the full reciprocal information required for voxel reconstruction. The virtual reciprocal scanning scheme was validated by a simulated 6D wide-angle X-ray diffraction tomography experiment. The theoretical validation of the method represents an important technological advancement for 6D diffraction tensor tomography and a crucial step towards pervasive applications in the characterization of heterogeneous materials.

## Abstract 2

A novel approach is reported for 6D wide-angle X-ray diffraction tensor tomography characterization based on the concept of virtual reciprocal-space scanning.

## 1. Introduction

The combination of X-ray scattering/diffraction techniques with computed tomography (CT) has a distinct advantage in simultaneously imaging a 3D structure and its functional properties (phase, shape, orientation and strain of the crystalline components), thus it has found broad applications in the study of lithium batteries (Jensen *et al.*, 2015 ▸; Finegan *et al.*, 2019 ▸), fibre-reinforced composites (Auenhammer *et al.*, 2024 ▸), catalysts (Sheppard *et al.*, 2017 ▸), metallic alloys (Stoica *et al.*, 2021 ▸) and biomaterials (Jensen *et al.*, 2011 ▸; Leemreize *et al.*, 2013 ▸). For powder or highly isotropic materials, the integration of a tomographic technique is rather straightforward as the signal is considered naturally rotational invariant (Feldkamp *et al.*, 2009 ▸; Schroer *et al.*, 2006 ▸). However, to characterize biological materials [bone (Rajasekharan *et al.*, 2018 ▸; Liu *et al.*, 2017 ▸; Grünewald *et al.*, 2023 ▸), tooth (Weng *et al.*, 2016 ▸), crustacean cuticle (Zhang *et al.*, 2017 ▸), *etc.*], which exhibit strong functional gradients and microstructural heterogeneity, the use of so-called small-angle X-ray scattering (SAXS) tensor tomography or wide-angle X-ray diffraction (WAXD) tensor tomography techniques are desired due to rotational invariance requirements.

These cutting-edge SAXS tensor tomography and WAXD tensor tomography techniques have attracted extensive attention in recent years for their unique potential in dynamic inspection of heterogeneous structures and mechanics. Compared with general SAXS/WAXD CT techniques, the volumetric reconstruction of SAXS tensor tomography and WAXD tensor tomography usually needs to collect 6D information across real and reciprocal space to satisfy rotational invariance. One of the earliest experimental demonstrations for 6D SAXS tomography was carried out at the cSAXS beamline of the Swiss Light Source by Schaff *et al.* (2015 ▸). By introducing a ‘virtual tomography axes’ scheme, which can take full advantage of the recorded scattering data and significantly reduce points for scanning information across the reciprocal space, they managed to characterize the 3D collagen fibre distributions in a millimetre-size human-tooth sample at a voxel size of 50 µm. Liebi *et al.* (2015 ▸), Gao *et al.* (2019 ▸ and Nielsen *et al.* (2023 ▸) further optimized the reconstruction algorithms to accelerate the data-intensive analysis process. All these works required at least 4D scans to obtain sufficient information for the final volumetric reconstruction of the scattering signals, which leads to an acquisition time of tens of hours. This therefore becomes the major limiting factor in practical usage. The situation will be worse in diffraction-based tensor tomography since a finer scan is needed to capture the faster reciprocal intensity variation of diffraction peaks compared with the scattering geometry. The radiation-induced damage is also a major concern due to the repetitive dose exposure in such multi-dimensional scan processes. While Zhou *et al.* (2023 ▸) effectively enhanced the signal-to-noise ratio of diffraction images acquired with low exposure times using deep-learning algorithms, which partially alleviates the problem caused by sample radiation damage, the fundamental issue remains unresolved. So far, WAXD tensor tomography experiments can only be applied to samples under strict constraints, such as measuring scattering predominantly in the direction of the tomography axis. Therefore, a better acquisition strategy is needed to address the problem of prolonged acquisition time for SAXS tensor tomography and WAXD tensor tomography methods.

As a matter of fact, the 3D scattering and diffractive reciprocal information is usually hidden in the 2D SAXS/WAXD pattern for most biological and synthetic materials exhibiting strong textures. In our previous study, we established a way to predict the 3D reciprocal information using a mathematical model for textured biological materials with fibre symmetry (the crystalline fibrils exhibiting rotational symmetry around the fibre axis) (Reiterer *et al.*, 1999 ▸; Lichtenegger *et al.*, 1999 ▸, 2003 ▸), and hence were able to determine the 3D fibre orientation via single-shot SAXS/WAXD images (Zhang *et al.*, 2016 ▸). This study showed that the (110) equatorial reflection of α-chitin in a WAXD pattern can be successfully used to quantitatively extract the 3D volumetric fibre orientation in the exoskeleton of a mantis shrimp.

In this article, we propose a novel approach for 6D WAXD tensor tomography characterization based on the concept of virtual reciprocal-space scanning: retrieving the 3D reciprocal information by mathematical modeling. A simulation study covering the entire workflow has been performed to demonstrate the feasibility of our method. The results indicate that combining mathematical modeling with a conventional 3D scanning tomography experimental setup is sufficient to reconstruct the 3D diffraction information in reciprocal space corresponding to each sample voxel and to resolve the 3D fibre orientation distribution within a 3D nanofibre-based sample. In particular, we propose a simple distributed scheme for reconstructing the 3D reciprocal intensity in each voxel of the sample. The method can be easily further accelerated by parallel computing to solve one of the most extreme tasks in data processing of future synchrotron tomography methods in terms of computational complexity, which involves reconstructing the total 6D information in real and reciprocal space. The new method is therefore a major step forward in terms of enabling fast data acquisition and facilitating *in situ* applications for 6D WAXD tensor tomography.

## 2. Methods

### 2.1. Virtual reciprocal-space scan strategy

The concept of retrieving the hidden 3D reciprocal information from 2D WAXD patterns using mathematical modeling has been demonstrated successfully in 2D raster scans on slices of stomatopod cuticle (Zhang *et al.*, 2016 ▸). Due to fibre symmetry, the rotation of the α-chitin unit cell around the *c* axis will lead to rings of equal intensity in the (110) reciprocal sphere, and the spreading of the nanofibres in 3D space will result in different diffraction intensity distribution patterns on the reciprocal spheres, which can be described using a mathematical model (Fig. S1 of the supporting information). The 3D nanofibre orientation parameters can then be extracted by fitting the azimuthal integration profile of the (110) diffraction generated using the mathematical model on the experimental data. However, the robustness and scalability of integrating the method into a 6D WAXD tensor tomography dataset has yet to be evaluated. Compared with the 2D raster scan experiment, in tomography experiments, the fibre geometry tends to be more arbitrary, since the angle between the incident X-ray beam and fibres within the same voxel continuously changes due to sample rotation. Therefore, the model needs to remain effective to retrieve sufficient information from reciprocal space across different fibre diffraction geometries.

**Figure 1:**
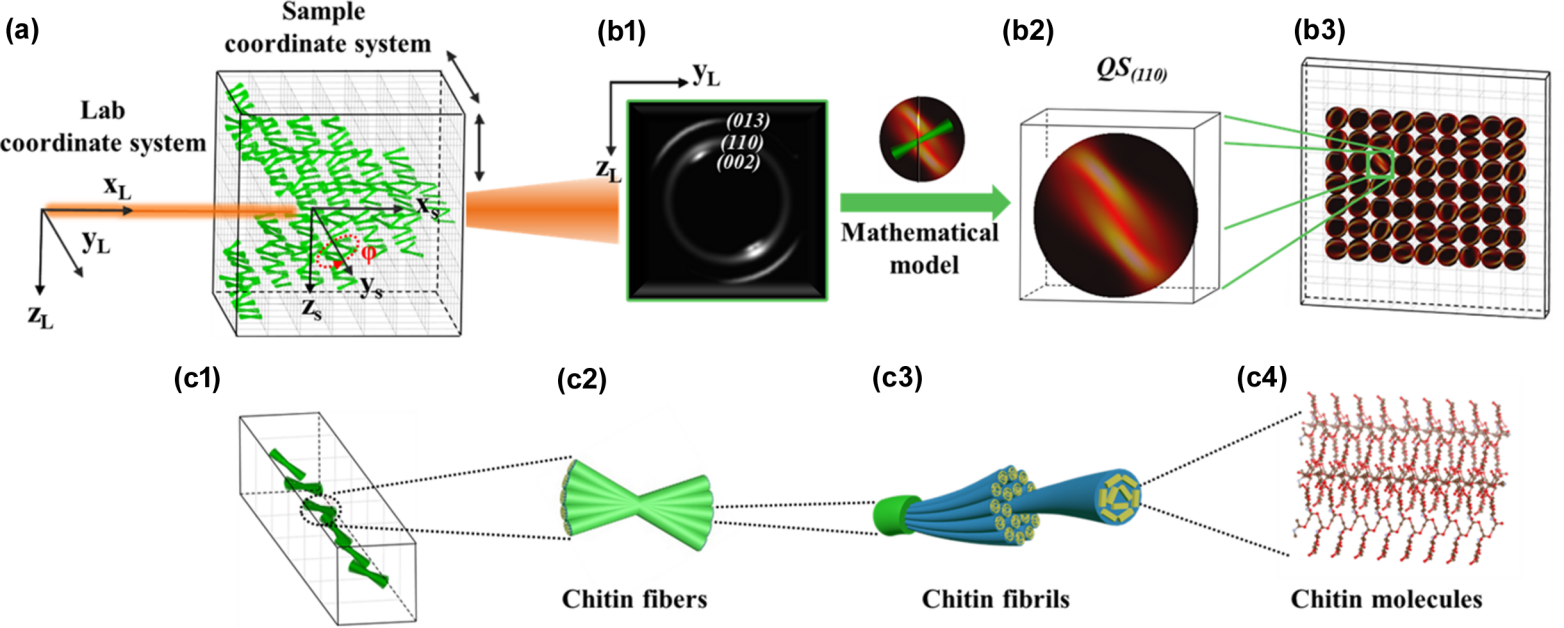
A schematic representation of the 6D WAXD tensor tomography scanning setup. (*a*) The detector coordinates and the 3D fibre orientation are described in the laboratory coordinate system. The sample is placed in the incident path (along the *x*_L_ axis) of the focused X-ray probe. The data acquisition only requires a regular scanning tomography protocol including a raster scan in the *y*_L_–*z*_L_ plane and sample rotation along the *y*_L_ axis. (*b*1) A 2D WAXD pattern is acquired at each scanning point, which is further used to retrieve (*b*2) the (110) reciprocal sphere [QS(110)] corresponding to the illuminated volume in the X-ray path. As a result, (*b*3) a 2D QS(110) array corresponding to the scanning projection (φ) is obtained for the subsequent voxel reconstruction. (*c*1)–(*c*4) Schematics indicate that the simulated sample exhibits similar lattice structure and fibre-symmetry features as mantis shrimp cuticles.

For method validation, we simulated a heterogeneous nanofibre-based sample structurally similar to the stomatopod cuticle, with the fibre orientation distribution deliberately randomized compared with the highly ordered texture in real stomatopod cuticles. Assuming the nanofibre exhibits the same fibre-symmetry feature and lattice structure as the chitin nanofibre in real cuticles [Fig. 1 ▸(*c*)], the (110) diffraction pattern of the nanofibre was employed to retrieve the reciprocal information using the mathematical model published by Zhang *et al.* (2016 ▸). As shown in Fig. 1 ▸(*a*), the data-acquisition process for our approach has no difference compared with conventional scanning tomography methods. A 2D raster scan (*y* and *z* axis) is conducted to collect WAXD patterns in each projection of the sample and then additional sample rotations (φ) are performed. In theory, the intensity distribution on the (110) reciprocal sphere [QS(110)] generated from each scanning point will be the accumulation of QS(110) spheres from all voxels within the X-ray illuminated volume along the beam path. Therefore, the (110) diffraction patterns captured on the detector will be a slice of the accumulated QS(110) sphere on the detector plane. For convenience, we assigned a specific index *j* to each voxel (*V_j_*) within the simulated sample regardless of rotation geometries.

### 2.2. Simulating a 6D WAXD tensor tomography dataset

**Figure 2:**
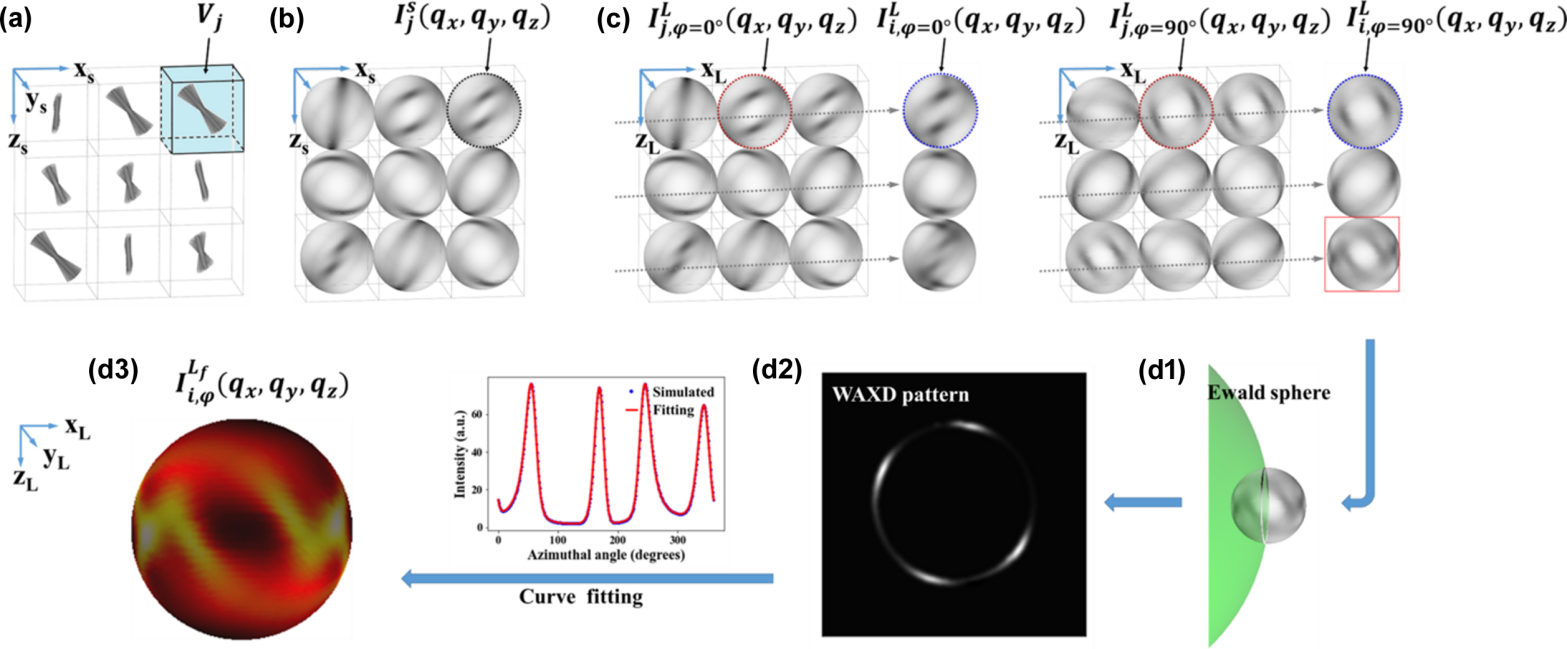
Schematics indicate the full simulation process to obtain the QS(110) generated at each scanning point. For simplification, only 3 × 3 voxels are shown here to represent the full sample. (*a*) Each sample voxel (*V_j_*) contains a group of nanofibres of certain 3D orientations, which leads to (*b*) 3D intensity distribution of corresponding 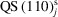 reciprocal spheres in the sample coordinate system. (*c*) The obtained 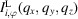 for each scanning point varies due to the accumulation of different voxel combinations along the beam path *i* at different rotation angles φ (left: φ = 0°; right: φ = 90°). 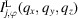 represents the 3D intensity distribution of 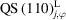 for each voxel in the laboratory coordinate system. (*d*2) A simulated 2D WAXD pattern of (110) reflection is acquired from the intersection plane between the accumulated 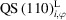

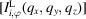
 and (*d*1) the Ewald sphere. (*d*3) The full 3D intensity distribution 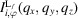 on the 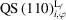 of each scanning point is retrieved via a fitting process using our mathematical model.

Fig. 2 ▸ describes the generation of each WAXD pattern of (110) reflection at different scanning points during the simulated tomography experiment, as described in the *Methods*[Sec sec2]. Based on the initial nanofibre orientation parameters in each sampling voxel, the 3D intensity distributions on the 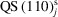 of each sample voxel (*V_j_*) [Fig. 2 ▸(*a*)] in the sample coordinate system can be described as 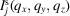 [Fig. 2 ▸(*b*)] according to the mathematical model. The 3D intensity distribution of each voxel in the non-sample area is set to zero. For each arbitrary rotational angle (φ), the 3D intensity distributions on the 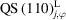 of each *V_j_* in the laboratory coordinates can be described as 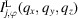, which is equal to the product of 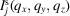 with matrix *R*_*y*_ [equation (3) in Appendix *A*[App appa]].

Then, 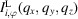 generated from each scanning point will be the accumulation of 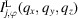 from all illuminated voxels along the X-ray beam path [Fig. 2 ▸(*c*)]. The effect of absorption on the diffraction signal is also considered and explained in the *Rotational invariance check* subsection in Appendix *A*[App appa]. A simulated WAXD pattern is further generated based on the specific diffraction geometry at each scanning point [Figs. 2 ▸(*d*1) and 2 ▸(*d*2)].

### 2.3. Reconstruction of the 3D reciprocal information from the 2D WAXD patterns

The first data-analysis step will be to retrieve the full 3D reciprocal information corresponding to each WAXD pattern. As in certain incident geometries, the fibre orientation of each voxel changes vastly along the X-ray beam path and therefore requires multiple groups of input orientation parameters for fitting. The retrieval process [Fig. 2 ▸(*d*3)] of 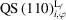 from the simulated 2D WAXD pattern in this article has been optimized by comparing it with the protocol described by Zhang *et al.* (2016 ▸) that deals with a fitting task involving multiple fibre groups. To find the optimal fitting orientation parameters of fibres, a basin-hopping algorithm (Li & Scheraga, 1987 ▸; Wales & Doye, 1997 ▸; Wales & Scheraga, 1999 ▸) was used to find the optimal solution within the constraints of parameters. Through the above process, a retrieved 3D intensity distribution of 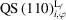 [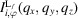] of each projection is acquired for further voxel reconstruction.

### 2.4. Distributed reconstruction scheme for voxel reconstruction

**Figure 3:**
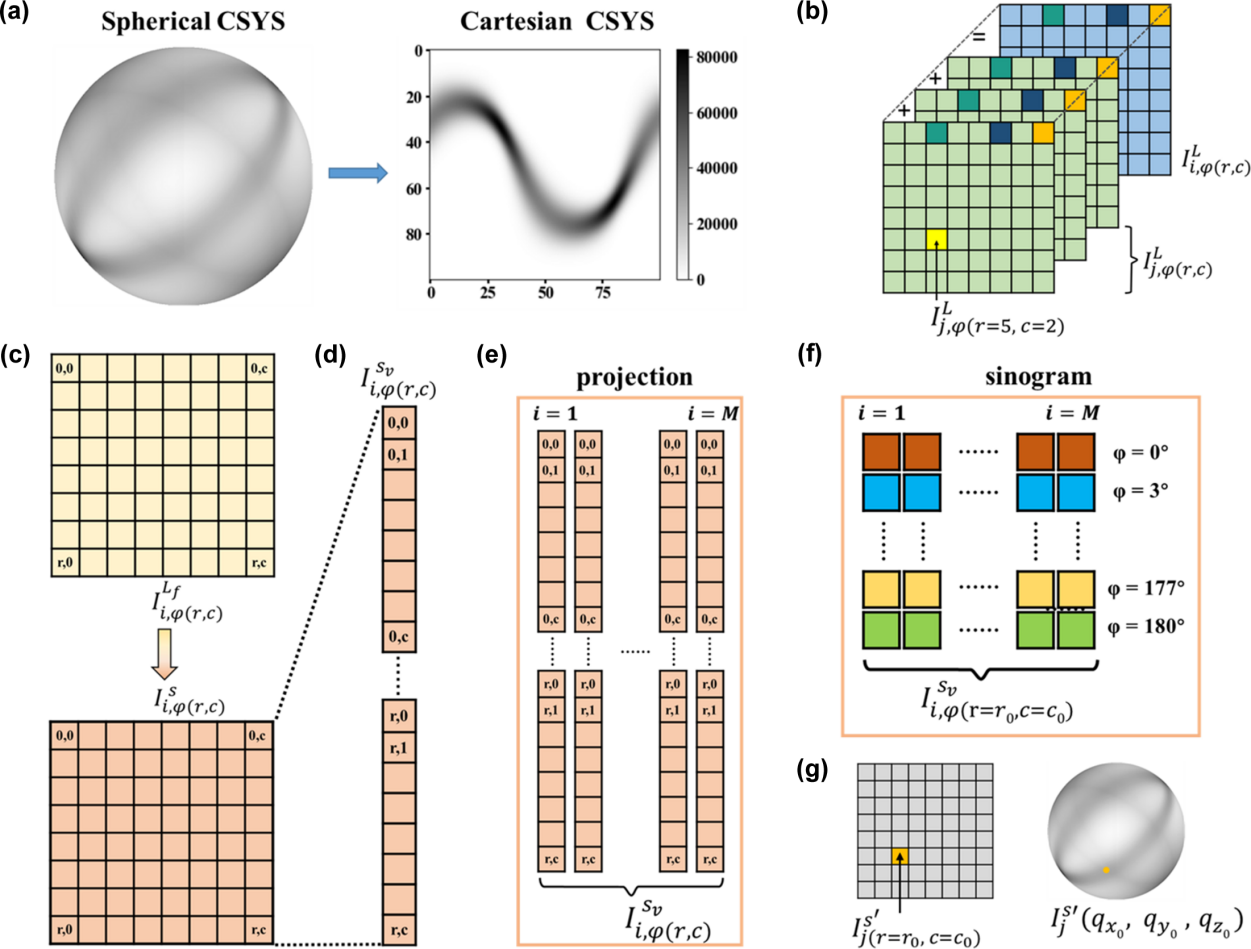
Schematics indicate the 6D tomography reconstruction process. (*a*) Transformation between the spherical coordinate system and the Cartesian coordinate system (CSYS). (*b*) A schematic representation of the 2D matrices in the X-ray path and the sum of those matrices at the same position (*r*, *c*). (*c*) A matrix-transformation process is performed on 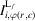 to get 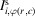 to fulfill rotational invariance. (*d*) The 2D intensity map [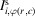] is then reshaped into a list of nodes with intensity indicated as 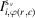, with each node assigned a 2D index of (*r*, *c*). (*e*) A projection with *M* raster scanning points will generate intensity maps for *M* columns. (*f*) Sinograms of each specific node (*r*_0_, *c*_0_) on the retrieved and transformed 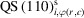 maps are extracted for the next paralleled reconstruction step. (*g*) The nodes on the reconstructed 2D intensity maps [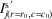] are used to assemble the final 3D 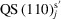 spheres [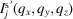] for each voxel.

A novel data-analysis scheme is proposed for the voxel reconstruction of a 6D real and reciprocal tomography dataset. To facilitate a subsequent reconstruction process, we transform the intensity-distribution expression of the reciprocal QS(110) sphere from the 3D spherical coordinate system to the 2D Cartesian coordinate system, as shown in Fig. 3 ▸(*a*). The 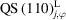 sphere of a certain voxel can therefore be represented by a 2D matrix with *r* rows and *c* columns, marked as 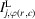, with a total number of *rc* nodes. The terms *r* and *c* are not necessarily equal. The sum of matrices [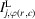] with respect to each 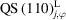 sphere from voxels in the X-ray path will be 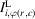 [Fig. 3 ▸(*b*)].

One condition that must be satisfied in tomographic reconstruction is the rotational invariance of the acquired signal. Since the orientation distribution of the nanofibres across the sample is anisotropic, the 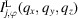 and 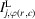 of 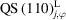 in each voxel change as the sample rotates in the laboratory coordinate system. Therefore, the sum of 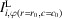 for all of the path [that is, 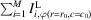] is not a constant, which obviously does not satisfy the rotational-invariance condition. However, the spherical intensity distribution of 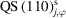 in each voxel remains the same in its sample coordinate system. An inverse matrix transformation is performed on 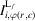 for the final voxel reconstruction, thus we obtained 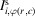, and the validation of its rotational invariance is described in detail within Appendix *A*[App appa]. For the multi-dimensional reconstruction process, each node on the 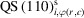 map [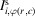] will be reconstructed independently using the standard filtered back-projection method (Palenstijn *et al.*, 2011 ▸; van Aarle *et al.*, 2016 ▸) following the procedure described in Figs. 3 ▸(*d*) to 3 ▸(*g*). In the end, a reconstructed 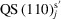 sphere [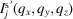] is assembled from reconstructed 2D intensity maps [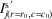] according to the node index.

## 3. Results

**Figure 4:**
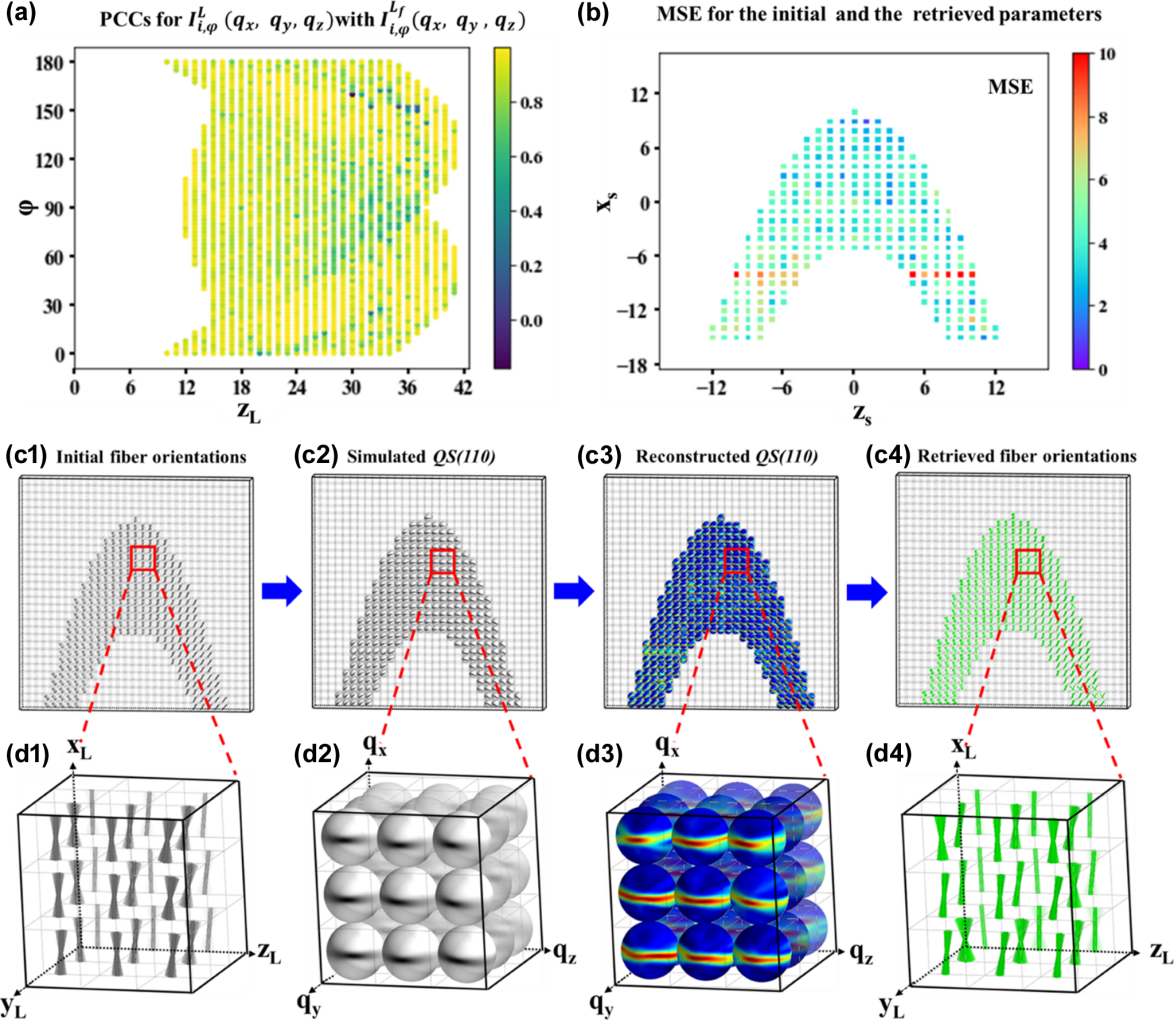
Comparison between the simulated sample and the reconstructed sample via proposed 6D WAXD tensor tomography. (*a*) PCCs between simulated and retrieved QS(110) spheres for a certain sinogram of one slice. (*b*) The combined MSE of the four orientation parameters between the initial and retrieved fibres for each voxel. (*c*1), (*c*2) A display of the initial fibre orientations in the simulated sample and corresponding 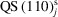 spheres in each voxel. (*c*3) Reconstructed 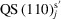 spheres representing each voxel. (*c*4) A display of retrieved 3D nanofibre orientations in each voxel. (*d*1)–(*d*4) Enlarged views of selected areas (3 × 3 × 3 voxels) from (*c*1)–(*c*4).

Sphere-to-sphere Pearson correlation coefficient (PCC) calculation between the simulated 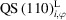 sphere and the retrieved 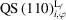 sphere via mathematical modeling helps quantify the retrieving accuracy [Fig. 4 ▸(*a*)]. The proportion of PCC values exceeding 0.8 for all scanning points among the full simulated tomography dataset is 89.36%, which means that almost all the 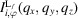 show a very high correlation with 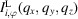. The results demonstrate the optimized mathematical modeling method and show great robustness regardless of arbitrary diffraction geometries during the rotational tomography scans. The fitting process for reconstructing the spherical intensity distributions on the 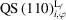 reciprocal sphere is the most computer-intensive task in the whole data-processing pipeline of the 6D WAXD tensor tomography method. There were 5874 out of a total of 9855 scanning points in which the X-ray beam illuminated the sample region in the simulated experiment. In order to fit those 5874 *I*(χ) curves, a total of 42 000 h was needed to run the whole fitting process on a single CPU core [Intel(R) Xeon(R) Gold 6348] originally. However, the time consumption was significantly reduced to 306 h by parallelization of the algorithms and using one GPU [Intel(R) Xeon(R) Gold 6348, A10, 80 GB] of the same model. The number of scanning points in future experiments will increase by about one to two orders of magnitude compared with our simulation test, the processing efficiency of the highly parallelized fitting algorithms developed in the current study can be easily scaled up using high-performance-computing facilities to handle the rising challenges. The retrieved 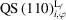 arrays are then used for further voxel reconstruction, as described in Figs. 3 ▸(*c*) to 3 ▸(*g*). Then, 400 × 400 meshes are generated on each retrieved 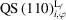, leading to a total of 160 000 sinograms for reconstruction corresponding to each mesh, which costs about 40 min on a single CPU core. The distributed reconstruction scheme can be naturally sped up by parallel computing techniques in the future. Within the simulated cuboid region, 942 out of a total of 1950 voxels are occupied with the sample, and the fibre-orientation parameters need to be extracted from these reconstructed [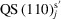] reciprocal spheres [Figs. 4 ▸(*c*3) and 4 ▸(*c*4)]. The orientations of the nanofibre in all voxels are extracted from the 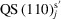 sphere intensity map using the Powell algorithm (Powell, 1964 ▸). Using the CPU, this process takes about 19 880 h. After implementing GPU parallel processing, the fitting time was reduced to about 27 h. The mean square error (MSE) was utilized to provide a quantitative comparison between the initial and retrieved fibre-orientation parameters. The MSE values for α_0_, β_0_, γ_0_ and Δγ_0_ are 2.09, 5.78, 5.24 and 4.22°, respectively. Fig. 4 ▸(*b*) illustrates the combined MSE of the four orientation parameters for each voxel (individual-parameter MSE is provided in Fig. S4). Approximately 78% of the voxels have an MSE of less than 5, indicating good agreement of fibre orientations with the initial values. Additionally, one row of voxels exhibited a larger MSE, possibly due to significant changes in the initial parameters of the fibre orientation [Fig. S2(*b*)]. In general, the fibre-orientation distributions within the reconstructed and simulated samples show acceptable consistency [Figs. 4 ▸(*d*1) and 4 ▸(*d*4)].

## 4. Conclusions

In conclusion, we have established a new method to accelerate the data acquisition of the cutting-edge 6D WAXD tensor tomography experiment. This technology, which employs mathematical models to extract the 3D information in the reciprocal space hidden in a 2D WAXD pattern, can obtain sufficient information for reconstructing 3D fibre-orientation distributions in each sample voxel with the same experimental setup as conventional scanning tomography. The reconstruction of the 3D reciprocal information using mathematical models is therefore equal to a virtual scan in the reciprocal space. The feasibility of the method was assessed by a simulation experiment. A highly distributed and parallel data-processing workflow was designed to handle the challenging data-analysis task for the proposed method. The results show that the reconstructed reciprocal sphere and fibre-orientation parameters for each voxel are both highly consistent with the initial settings, which demonstrates the feasibility and effectiveness of our method. The theoretical verification of our method represents a crucial step forward for the technological advancement of SAXS/WAXD tensor tomography.

Our method can significantly improve the data-acquisition efficiency by reducing the scan procedure with at least one degree of freedom; therefore, benefitting *in situ* characterization and radiation-sensitive sample studies. In the next phase, we will focus on addressing the algorithmic and software challenges resulting from the big data stream in the acquisition and analysis stages (Zhang *et al.*, 2023 ▸) in order to implement the method in the beamlines. To further reduce the radiation damage, powerful denoising techniques need to be developed to implement on the acquired WAXD images for enhancing signals of interest, which helps obtain sufficient diffraction information with minimum X-ray dose. Furthermore, the replacement of traditional reciprocal-sphere reconstruction methods with machine-learning methods has great potential to speed up the entire data-analysis pipeline and increase accuracy (Sun *et al.*, 2023 ▸). Together with the advances in beam brilliance, instrumentation, experimental control, data acquisition and analysis software (Dong *et al.*, 2022 ▸; Liu *et al.*, 2022 ▸; Zhang *et al.*, 2024 ▸) in the next-generation synchrotron beamlines, we expect to push the 6D WAXD tensor tomography technique to the limit to enable its prevailing use in structural and mechanical study of heterogeneous materials.

## 5. Data availability

All Python codes that support the findings in this study are available from the corresponding authors upon request.

## Supplementary Material

Supporting information. DOI: 10.1107/S2052252524003750/fs5219sup1.pdf

## Appendix A

### A1. Formula superscript and subscript explanation

: the simulated 3D intensity distribution of  in the sample coordinate system, where ‘s’ refers to the sample coordinate system and *j* refers to the position number of voxels.

: the reconstructed 3D intensity distribution of  in the sample coordinate system.

: the 3D intensity distribution of  at a certain X-ray incidence path in the laboratory coordinate system, where L refers to the laboratory coordinate system, *i* refers to the number of the X-ray incidence path and φ refers to the rotation angle.

: the 3D intensity distribution of  retrieved from 2D WAXD patterns using our mathematical model.

: the 3D intensity distribution of  after inverse matrix transformation is applied on .

### A2. Sample and diffraction data-acquisition simulation

A nanofibre-based simulation sample is designed to mimic the central carina region of the cross section of mantis shrimp telson examined in the previous study (Zhang *et al.*, 2016 ▸). A cuboid with a size of 3(*y*) × 26(*x*) × 25(*z*) voxels is required to fully frame the sample. To prevent the sample from falling out of the field of view, a raster scan of 45(*z*) × 3(*y*) points at a step size equal to the side length of a voxel was performed at each projection. A total of 73 projections were collected from 0 and 180° around the *y* axis at a step size (Δφ) of 2.5°. A total of 9855 QS(110) spheres were generated for the simulation experiment.

To simplify the simulation, we assume that there is only one group of nanofibres inside each voxel with its 3D orientation parameters defined as (α_0_, β_0_, γ_0_, Δγ_0_), where Δγ_0_ is defined as the width parameter of the fibre distribution. However, the voxel can be partially or fully illuminated by the incident beam due to the rotation of the sample. When calculating  for a specific scanning point, we introduce a weight factor *w*_*ij*_ to represent the contribution of each voxel in the sample.

Whether the voxel is located in the illuminated volume or not can be easily deduced by simple geometric calculations. Assume that the nanofibres [represented by a dot in Fig. S2(*a*)] are placed in the center of each voxel and the width of the X-ray beam is equal to the side length of a voxel, if the dot of the voxel falls in the X-ray path, the *w*_*ij*_ of the voxel is assigned to be 1. Otherwise, the *w*_*ij*_ of the voxel is 0. At any rotation angle, the scanning process of the sample is equivalent to the translation of the X-ray beam. Fig. S2(*a*) shows the contribution of each nanofibre in the X-ray incident path during the beam translation.

The sample-absorption effect on the diffraction signals has also been taken into consideration. For simplification, we assume that the absorption attenuation coefficient of each sample voxel is the same, denoted as μ. To simulate the previous WAXD experiments on stomatopod cuticle, we set the absorption coefficient to be μ = 5.30 cm^−1^, and assume that the sample is examined with X-ray energy of 13 keV and mainly made of calcium carbonate. The attenuation effect on the acquired diffraction signal is therefore determined by the sample volume along the beam path. We also introduce a variable *D*_φ,*ij*_, which is related to the distance that the diffracted signal of voxel *j* travels on path *i* at rotation angle φ.

Thereby, in our model, linear equations were used to describe the 3D intensity distribution in the X-ray path at rotation angle φ:

where *M* represents the sampling numbers in the *z*_L_-axis direction (in our model, *M* = 45), *N* = 650,  is the 3D intensity distribution on the  corresponding to each voxel in rotation angle φ in the laboratory coordinate system,  is the 3D intensity distribution corresponding to the sum of  in the *i*th X-ray path (*i* − 1 ≤ *z*_L_ < *i*) at rotation angle φ, the weight factors *w*_*ij*_ (0 or 1) represent the contributions of a voxel *j* on the diffraction in the *i*th X-ray path, μ is the absorption attenuation coefficient of each voxel and *D*_φ,*ij*_ is related to the distance the diffracted signal of voxel *j* travels on path *i* at rotation angle φ.

We set an initial parameter for nanofibre orientation as (α_0_, β_0_, γ_0_, Δγ_0_) for each voxel in one of the sample slices (*z*_s_–*x*_s_ plane) at the sample coordinate system according to Fig. S2(*b*). Since our purpose is to verify the feasibility of 6D WAXD tensor tomography using virtual scanning, it is sufficient for us to choose a slice in the *z*_s_–*x*_s_ plane and set initial parameters of nanofibre orientation in this way.

### A3. 3D reciprocal-space data acquisition of diffraction signals

A basin-hopping algorithm was used to find the global optimal solution in the curve-fitting process. As shown in Fig. S3(*b*), by using three groups of fibre parameters (*a* = 1, 2, 3), the goodness of fit (*R*^2^) between *I*(χ) curves of simulated and fitted WAXD patterns collected at a scanning point can reach 99.1%, and the PCC of a simulated  sphere [Fig. S3(*a*)] and a retrieved  sphere [Fig. S3(*c*)] is 0.91.

### A4. Data processing

The simple process of experiment simulation and the data-processing procedure are briefly given in Fig. S5.

### A5. Rotational-invariance check

A projection-dependent rotation matrix *R*_*y*_ [equation (1)] is used for the transformation between the sample coordinates  and the laboratory coordinates :

in which *R*_*y*_ is

The 3D intensity distribution of QS(110) from a scan point can be expressed as

An inverse matrix is then applied on  to get the accumulated QS(110) on the sample coordinate system over the voxels in the illuminated path at a certain scan point, denoted as :

The  is further converted into  in Cartesian coordinates:

Since  and the corresponding  are constant in the sample coordinate system,  in all X-ray paths at rotation angle φ is equivalent to the sum of  corresponding to all voxels in the sample coordinate system, which means that the projection formed by the same position (*r*, *c*) of  is rotation invariant.
