# Supplementary material for: A step towards 6D WAXD tensor tomography
Source: IUCrJ. 2024 May 9;11(Pt 4):502–9. doi: 10.1107/S2052252524003750 (PMC11220869; doi:10.1107/S2052252524003750)
Supplement: Supplementary file 1 [file m-11-00502-sup1.pdf]

IUCrJ

Volume 11 (2024)

Supporting information for article:

A step towards 6D WAXD tensor tomography

Xiaoyi Zhao, Zheng Dong, Chenglong Zhang, Himadri Gupta, Zhonghua Wu, Wenqiang Hua, Junrong Zhang, Pengyu Huang, Yuhui Dong and Yi Zhang

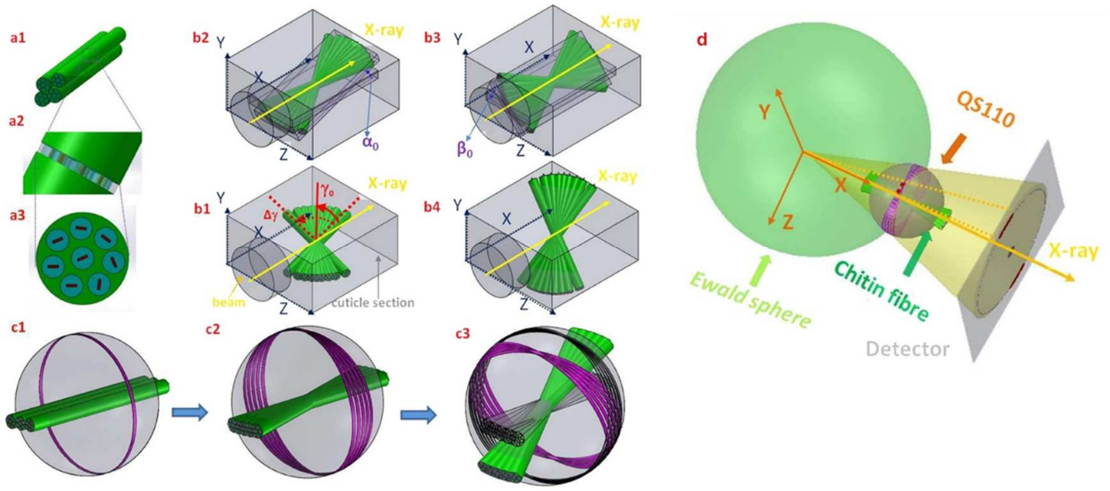

Figure S1 Schematic showing the fiber symmetry and how the 3D distribution of nanofibers affect the diffraction intensity distribution on *(110)* reciprocal spheres. Each nanofiber (a1) are composed of many nanofibrils exhibiting rotational symmetry around the nanofiber axis (a3). The randomly oriented reciprocal *(110)* vectors from each single fiber result in smearing of reflections into reflection rings in the *QS(110)*, i.e. (c1). Schematics illustrating the in-plane fiber orientation distribution parameters (γ0, Δγ0) within a single lamella plane parallel to the beam (b1). The orientation spreading of nanofibers in-plane will result in a reflection band on the sphere (c2). The yellow arrow in b1 denotes the direction of the incident beam. The out-of-plane tilt of the nanofiber planes in 3D (α, b2), (β, b3) or a combination of both angles (b4) will lead to the tilting of the reflection band (c3) on *QS(110)*. (d) Sketch of the diffraction geometry in experiments, with the Ewald's sphere shown on left and *QS(110)* on right. The intensity on the intersection ring of the Ewald's sphere and *QS(110)* will be collected as the experimental *(110)* diffraction pattern. Note this image is a reproduction of the Figure 2 in previous paper (Zhang *et al.*, 2016)

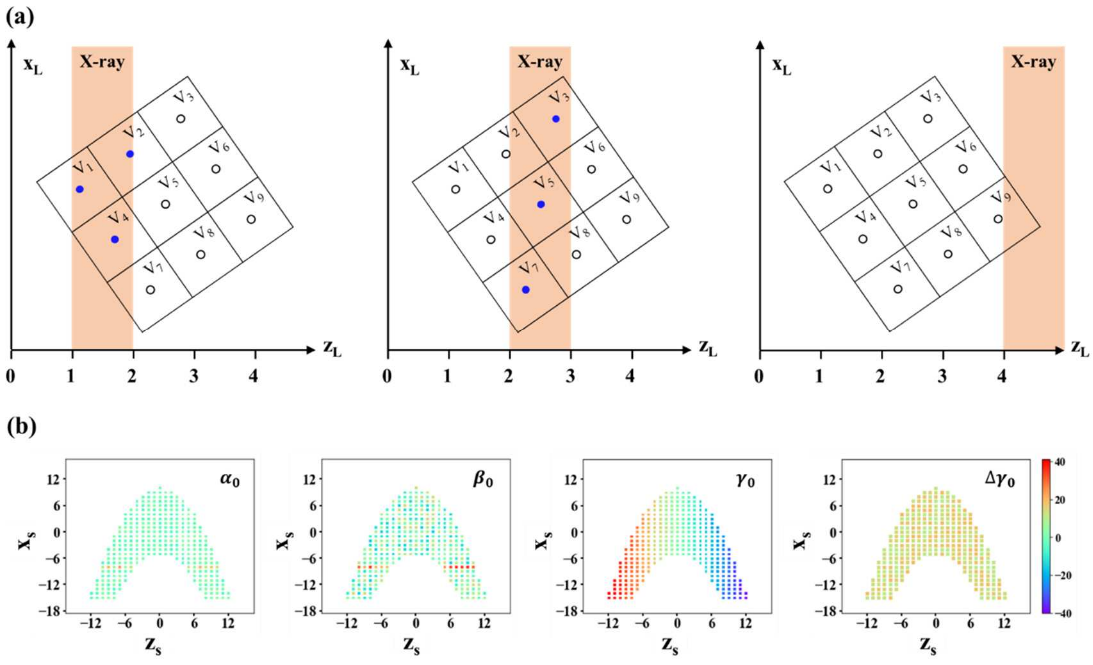

Figure S2 (a) Schematic representation of the weight factor in the X-ray path during sample (3*3 voxel) translation. The blue dots in the X-ray path represent that the w_{ij} of the voxels corresponding to the blue dots is 1, others are 0. (b) The initial parameter of the nanofibers orientation for one slice of the sample. The colored area is the sample area, and the blank area is not the sample, thus 25*26 voxels in total are required to fully frame the sample.

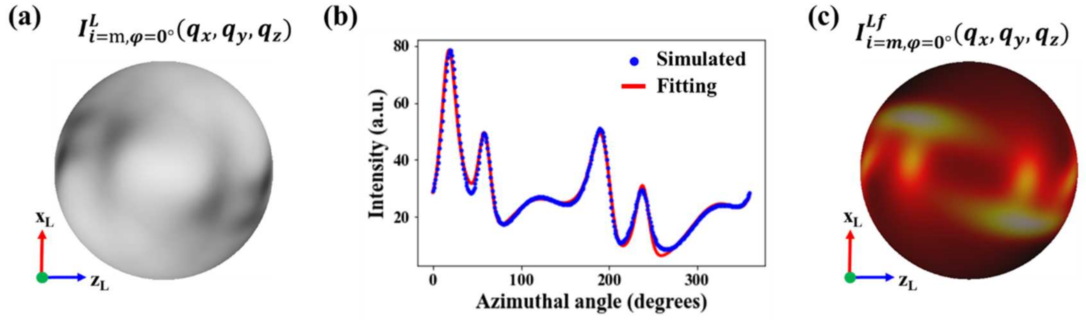

Figure S3 **The comparison between the simulated *QS(110)* and the retrieved *QS(110)* using mathematical modelling**. (a) The simulated $QS(110)^{L}_{i,\varphi}$ sphere based on a combination of voxels in the beam path at a specific scanning point; (b) The azimuthal integration curve *I(χ)* corresponding to the simulated $QS(110)^{L}_{i,\varphi}$ sphere and the fitted *I(χ)* curve; (c) The retrieved $QS(110)^{Lf}_{i,\varphi}$ using proposed mathematical modelling method.

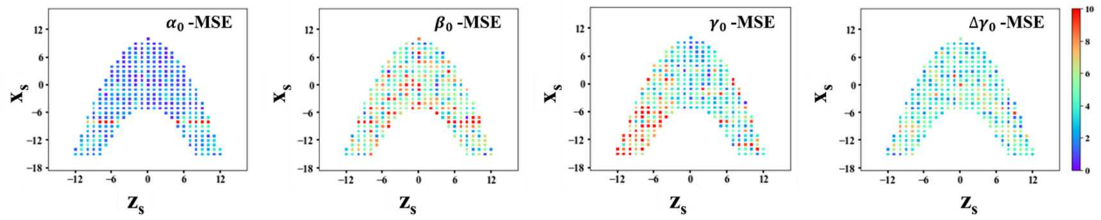

Figure S4 The mean square error (MSE) between the initial and the retrieved parameters of the nanofiber orientation for each voxel.

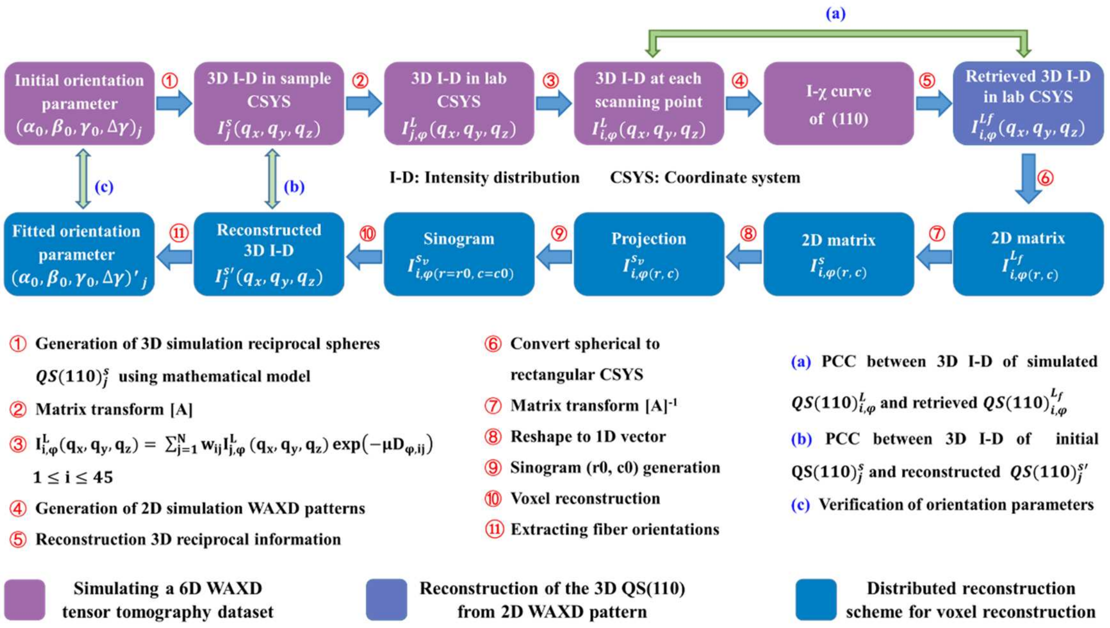

Figure S5 The schematic representation of the simulation process from tensor tomography dataset to reconstruction.

References

Zhang, Y., Paris, O., Terrill, N. J. & Gupta, H. S. (2016). *Scientific reports* **6**, 26249.
